# Green-Synthesized Rutin-Capped Gold Nanoparticles Attenuate Experimental Liver Fibrosis by Targeting Oxidative Stress and TGF-β Signaling

**DOI:** 10.3390/nano16060379

**Published:** 2026-03-22

**Authors:** Roxana Maria Decea, Ioana Baldea, Gabriela Adriana Filip, Luminita David, Bianca Moldovan, Vlad Toma, Claudia-Andreea Moldoveanu, Mara Muntean, Simona Valeria Clichici

**Affiliations:** 1 Department of Physiology, “Iuliu Haţieganu” University of Medicine and Pharmacy, Clinicilor 1, 400006 Cluj-Napoca, Romania; roxana.decea@elearn.umfcluj.ro (R.M.D.); sclichici@umfcluj.ro (S.V.C.); 2 Department of Anatomy and Embryology, “Iuliu Hațieganu” University of Medicine and Pharmacy, 8 Victor Babeş Street, 400012 Cluj-Napoca, Romania; afilip@elearn.umfcluj.ro; 3 Research Centre for Advanced Chemical Analysis, Instrumentation and Chemometrics, Faculty of Chemistry and Chemical Engineering, Babes-Bolyai University, 400347 Cluj-Napoca, Romania; bianca.moldovan@ubbcluj.ro; 4 Faculty of Medical and Health Sciences, Babes-Bolyai University, Clinicilor Str. No. 9, 400008 Cluj-Napoca, Romania; vlad.toma@ubbcluj.ro; 5 Institute of Biological Research from Cluj-Napoca, Branch of NIRDBS Bucharest, Republicii Str. No. 48, 400015 Cluj-Napoca, Romania; claudia.moldoveanu@ubbcluj.ro; 6 Department of Molecular Biology and Biotechnology, Babes-Bolyai University, Clinicilor Str. No. 5-7, 400008 Cluj-Napoca, Romania; 7 Department of Cell and Molecular Biology, “Iuliu Hațieganu” University of Medicine and Pharmacy, 400349 Cluj-Napoca, Romania; muntean.mara@elearn.umfcluj.ro

**Keywords:** gold nanoparticles, rutin, thioacetamide, liver fibrosis, TGF-β, oxidative stress, glutathione redox balance, pro-inflammatory cytokines

## Abstract

Liver fibrosis is driven by persistent oxidative stress and inflammatory signaling, with transforming growth factor-β (TGF-β) acting as a key profibrotic mediator. Rutin (Ru) is a plant-derived flavonoid with antioxidant and anti-inflammatory effects, but its low bioavailability limits therapeutic efficacy. This study investigated whether rutin-phytoreduced gold nanoparticles (RuAuNPs) enhanced rutin delivery leading to antifibrotic and anti-inflammatory effects in a rat model of liver fibrosis. Liver fibrosis was induced by oral administration of thioacetamide (TAA, 150 mg/kg body weight, p.o.) for six weeks. Following fibrosis induction, the animals were treated with free rutin (30 mg/kg body weight), RuAuNPs (0.3 mg/kg body weight), or AuNPs (0.3 mg/kg body weight), both expressed as nanoparticle mass, all administered orally for four weeks. RuAuNPs were synthesized by green rutin-mediated reduction and further characterized by TEM, DLS, and FTIR spectroscopy; they were spherical, showing an average hydrodynamic size of 104.1 nm (PDI 0.345). FTIR confirmed rutin capping. Biological effects were evaluated by liver morphology (H&E histology, TEM), biochemical assessment of liver aminotransferases and glico-lipidic status, ELISA and spectrophotometry measurement of redox biomarkers (lipid peroxidation, glutathione status, antioxidant enzymes), cytokines (TNF-α, IL-1β, IL-6), and TGF-β. TAA-induced hepatic injury and remodeling with increased profibrotic signaling, oxidative stress, and inflammation. Free rutin slightly ameliorated the liver damage, whereas RuAuNP improved histological features, reduced TGF-β and pro-inflammatory cytokines, decreased lipid peroxidation, and supported antioxidant defenses. Overall, RuAuNP may enhance rutin efficacy in TAA-induced liver fibrosis, with novelty stemming from the integrated in vivo evaluation of tissue changes and key profibrotic/oxidative/inflammatory pathway.

## 1. Introduction

Chronic liver disease involves a progressive loss of hepatic function, impairing essential processes such as protein and coagulation factor synthesis, detoxification, and bile production [1]. It arises from ongoing cycles of inflammation, hepatocellular injury, and regeneration, which eventually overwhelm the liver’s regenerative capacity. This results in excessive extracellular matrix deposition, leading to liver fibrosis and, in advanced stages, cirrhosis [2,3]. Liver fibrogenesis is a wound-healing response to chronic injury aimed at maintaining tissue integrity and involves coordinated interactions among multiple cell types and molecular mediators [4,5]. Hepatic stellate cells (HSCs) are key drivers of this process. Normally quiescent, HSCs become activated following liver injury and transition into proliferative, myofibroblast-like cells that produce extracellular matrix components [5,6].

Reactive oxygen species (ROS) act as important pro-fibrotic signals by activating pathways such as transforming growth factor-β (TGF-β), which promotes collagen synthesis and inhibits matrix degradation, and accelerate fibrosis progression [7,8,9].

Rutin is a naturally occurring flavonoid found in plants such as buckwheat, tea, and apples. It consists of quercetin linked to the disaccharide rutinose and exhibits notable antioxidant and anti-inflammatory activities. These effects are mediated through regulation of intracellular redox balance and inhibition of pro-inflammatory mediators, including TNF-α, IL-6, IL-1β, and COX-2, suggesting its hepatoprotective potential [10,11].

Gold nanoparticles (AuNPs) offer a promising strategy for antifibrotic therapy due to their favorable biodistribution and liver-targeting properties. Their nanoscale size promotes hepatic accumulation via the enhanced permeability and retention effect, with uptake primarily by Kupffer cells and hepatic stellate cells—key mediators of liver fibrosis [12]. Functionalized AuNPs have been shown to reduce hepatic stellate cell activation, including decreased α-smooth muscle actin expression, and to exert antioxidant and anti-inflammatory effects by suppressing cytokine production and oxidative stress [13].

Emerging evidence from in vivo and in vitro studies indicates that flavonoid-based nanomedicines, including quercetin-derived formulations, can attenuate liver fibrosis by improving redox balance and inhibiting profibrogenic signaling [14].

Consistent with the role of oxidative stress and inflammation in fibrogenesis, several antioxidant/anti-inflammatory interventions have been shown to attenuate TAA-induced liver fibrosis, often in parallel with reduced profibrotic signaling. For example, hesperidin ameliorated TAA fibrosis by antioxidative/anti-inflammatory actions with modulation of TGF-β/α-SMA [15], while naringin attenuated TAA fibrosis alongside reductions in oxidative stress and inflammatory mediators, with pathway modulation reported [16]. In this context, rutin’s pharmacological rationale is supported by its ability to modulate redox and inflammatory signaling, including Nrf2/HO-1 and NF-κB-related pathways, which are directly relevant to maintaining antioxidant and anti-inflammatory effects under ongoing hepatic injury [17]. Notably, rutin also showed antioxidant/anti-inflammatory activity in a non-hepatic inflammation model, reducing lipopolysaccharide (LPS)-induced acute lung injury via inhibition of oxidative stress and MAPK/NF-κB signaling, together with upregulation of antioxidative enzymes [18].

Based on this background, the rationale of the present study was to test whether delivering rutin via a bioinspired gold nanoparticle platform can enhance its functional antifibrotic effects in vivo. Therefore, we aimed to develop rutin-mediated gold nanoparticles (AuNPs) and to evaluate their efficacy in a validated thioacetamide-induced liver injury model previously established by our group, which reliably induces oxidative stress, inflammation, and fibrosis [19]. The model enables the assessment of the studied compound’s ability to modulate redox imbalance and TGF-β–driven profibrotic pathways, done by histopathological and biochemical analyses.

The novelty of this study is the in vivo evaluation of a rutin-based nano-delivery approach in a validated TAA-induced liver fibrosis model, with integrated assessment of histology/ultrastructure and key profibrotic (TGF-β) and inflammatory/oxidative pathways. By comparing free rutin with nanoparticle-mediated delivery, we address whether nanoformulation can enhance functional efficacy under fibrogenic injury conditions.

## 2. Materials and Methods

### 2.1. Preparation and Characterization of Rutin-Reduced Gold Nanoparticles

Rutin-reduced gold nanoparticles were synthesized through a green chemical reduction approach, using rutin as both a reducing and stabilizing agent. Briefly, 61 mg of rutin was dispersed in 100 mL of ultrapure deionized water, and approximately 4 mL of a 2 M aqueous sodium hydroxide solution was gradually added under continuous stirring until complete dissolution of rutin was achieved and the solution exhibited a yellow-orange coloration. To this basic rutin solution, 100 mL of a 1 mM aqueous solution of chloroauric acid (HAuCl_4_) was added, and the reaction mixture was stirred at 700 rpm for 2 h at room temperature to ensure complete reduction of Au^3+^ ions, allowing the formation of gold nanoparticles. The reaction mixture gradually changed color from pale yellow to ruby red, indicating the formation of gold nanoparticles due to the specific surface plasmon resonance of colloidal gold.

The synthesized gold nanoparticles were purified by centrifugation to remove unreacted rutin and soluble byproducts. The colloidal suspension was centrifuged using an Eppendorf 5425 R refrigerated centrifuge (Eppendorf, Leipzig, Germany) at 10,000 rpm, at 15 °C for 20 min, and the supernatant was discarded. The resulting pellet was dispersed in 10 mL deionized water using mild sonication (40 KHz ultrasonic waves), using a LBX Instrument ultrasonic bath (LABBOX Labware, Barcelona, Spain) of 3.2 L volume with an input current of 220–240 V and an ultrasound power of 120 Watt for 5 min. The washing process was repeated three times to ensure adequate purification.

The resulting pellet after centrifugation was resuspended in 10 mL of deionized water and stored at 4 °C until further characterization, application studies and biological experiments.

Formation of colloidal gold nanoparticles was monitored by UV–Vis spectroscopy, with absorption spectra recorded in the 300–800 nm range, using a PerkinElmer Lambda 25 UV–Vis spectrophotometer (PerkinElmer, Shelton, CT, USA). The formation of AuNPs was confirmed by the appearance of a characteristic surface plasmon resonance (SPR) band typically around 520–550 nm.

Transmission electron microscopy was employed to assess nanoparticles’ morphology and size, using a Hitachi H-7650 transmission electron microscope operating at 120 kV (Hitachi, Tokyo, Japan). TEM samples were prepared by placing a drop of the AuNP suspension onto a carbon-coated copper grid and allowing it to dry under ambient conditions.

The average diameter of the obtained nanoparticles was estimated from the TEM image by measuring the size of 100 randomly selected nanoparticles using ImageJ software, version 1.53t.

The hydrodynamic size and polydispersity index (PDI) of the nanoparticles in solution were determined by dynamic light scattering (DLS). The measurements were performed using a Malvern Zetasizer NanoSeries (Malvern Instruments, Malvern, UK) equipped with a laser source (633 nm) and a backscattering detection angle of 173°. All measurements were carried out at 25 °C.

Fourier-transformed (FTIR) spectra were recorded using a Bruker Alpha II spectrometer (Brucker Nano Gmb, Berlin, Germany) equipped with an attenuated total reflectance (ATR) accessory. The measurements were performed at room temperature, in the range of 4000–400 cm^−1^ with a resolution of 4 cm^−1^.

### 2.2. Biological Experiments

#### 2.2.1. Experimental Design

The study was conducted on 42 healthy adult female Wistar albino rats, aged 4 months and weighing 280 ± 20 g, obtained from the animal facility of “Iuliu Hațieganu” University of Medicine and Pharmacy, Cluj-Napoca. Animals were housed under controlled environmental conditions (12 h light/12 h dark cycle, 35% relative humidity, constant temperature), with ad libitum access to water and a standard normo-caloric diet. All experimental procedures involving animals were conducted in accordance with the ARRIVE guidelines and complied with national and international regulations for the care and use of laboratory animals. The study protocol was reviewed and approved by the Institutional Animal Care and Use Committee of “Iuliu Hațieganu” University of Medicine and Pharmacy and by the Veterinary Public Health Directorate of Cluj-Napoca (approval no. AVZ56/26.03.2024), and all efforts were made to minimize animal suffering and reduce the number of animals used.

The selected weight range (280 ± 20 g) reflects physiologically mature adult rats and was chosen to ensure adequate tolerance to the prolonged TAA exposure and to minimize variability related to ongoing growth. This range is consistent with our previously validated TAA-induced liver injury/fibrosis protocol and our group’s prior TAA/AuNP experimental settings [20,21].

**Figure 1:**
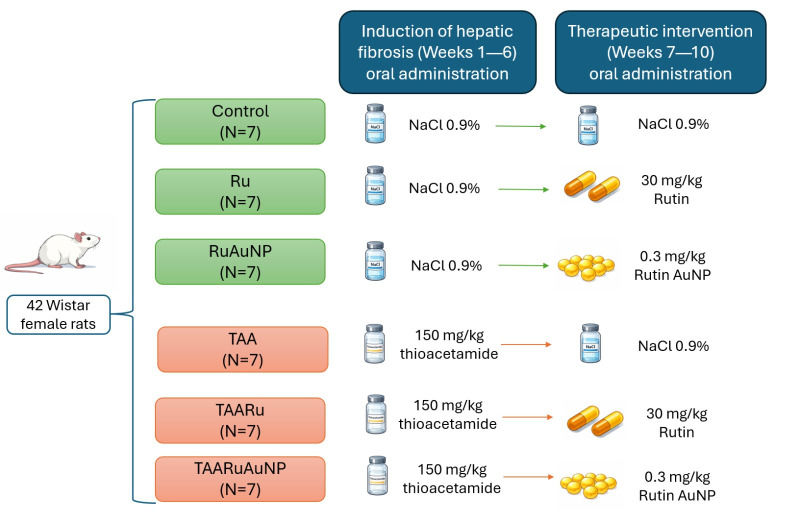
Experimental timeline of the fibrosis induction and therapeutic intervention.

Animals were randomly allocated into six experimental groups (*n* = 7/group). Physiological saline (0.9% NaCl) was used as a vehicle for all treatments. All formulations were prepared in 0.9% NaCl and adjusted to a final administration volume of approximately 0.5 mL per animal to ensure consistency across experimental groups (Figure 1). Groups I–III received 0.5 mL of physiological saline once daily for six consecutive weeks. In groups IV–VI, hepatic fibrosis was induced by oral administration of thioacetamide (TAA, 150 mg/kg body weight, diluted in saline) once daily for six consecutive weeks. Starting from the 7th week, animals received the following treatments, administered orally, once daily for four consecutive weeks: group I (Control)—vehicle (0.5 mL of 0.9% NaCl); group II (Ru)—rutin (30 mg/kg body weight); and group III (RuAuNP)—rutin-mediated gold nanoparticles (0.3 mg/kg body weight, nanoparticle mass); group IV (TAA)—vehicle (0.5 mL of 0.9% NaCl); group V (TAARu)—rutin (30 mg/kg body weight); and group VI (TAARuAuNP)—rutin-mediated gold nanoparticles (0.3 mg/kg body weight, nanoparticle mass). All treatments were administered for four weeks of the experimental protocol (Figure 1).

Physiological saline (0.9% NaCl) was used as a vehicle to minimize osmotic effects and potential mucosal irritation associated with hypotonic vehicles such as distilled water and to keep vehicle conditions consistent across groups. Control animals received saline by oral gavage in the same volume (0.5 mL/animal) to mimic the gavage procedure and to control for vehicle and handling effects. All treatments were adjusted to this final administration volume.

The rutin dose (30 mg/kg b.w.) was selected as a moderate, literature-supported oral exposure. The used dose was within the range commonly used in rat models to achieve antioxidant effects while avoiding excessive dosing. Rutin exhibited antioxidant and hepatoprotective effects, when administered orally at a dose of 25 mg/kg/day in a liver-toxicity setting [22] and respectively at 50–70 mg/kg in experimental hepatotoxicity models [23]. The 0.3 mg/kg dose corresponds to the administered AuNP (nanoparticle mass), rather than an equimass amount of free rutin. It was selected as a low, biologically active exposure based on our group’s previously validated TAA hepatopathy protocol using oral TAA (150 mg/kg for 6 weeks) and AuNPs (0.3 mg/kg), where antioxidant and anti-inflammatory effects were consistently observed [20].

At the end of the 10-week experimental period, animals were humanely euthanized by overdose of ketamine and xylazine, in accordance with accepted ethical standards for laboratory animal care. Blood samples were collected from the retro-orbital sinus into heparinized tubes and centrifuged at 3500 rpm for 10 min for plasma separation and further processed for biochemical analyses. Liver tissue samples were collected and used for the evaluation of inflammatory markers, oxidative stress parameters, morphology examination by histopathology and transmission electron microscopy (TEM) analyses.

#### 2.2.2. Histopathology Evaluation

Liver tissue samples were collected at necropsy and immediately fixed in neutral buffered 10% formalin. After adequate fixation, specimens were processed through graded ethanol dehydration and xylene clearing and were subsequently embedded in paraffin. Paraffin blocks were sectioned on a rotary microtome at a thickness of 5 µm. The sections were mounted on glass slides, dried, and subjected to routine hematoxylin–eosin (H&E) staining following standard histological protocols. Histological evaluation was performed by light microscopy. Necro-inflammatory activity and architectural alterations were assessed using the Knodell scoring system [24]. The components of the score, including periportal and bridging necrosis, intralobular degeneration and focal necrosis, portal inflammation, and fibrosis, were individually evaluated according to predefined criteria. For each sample, partial scores were summed to generate the total Knodell histological activity index. For each experimental group, individual scores were recorded and subsequently used to calculate a group index. Data were expressed as the distribution of Knodell scores within each group, and comparative analysis between experimental groups was based on these index values. All scoring was performed in a blinded manner to minimize observer bias.

#### 2.2.3. Transmission Electron Microscopy

The liver samples were fixed with glutaraldehyde (Agar Scientific Ltd., Stansted, UK) at 4 °C in), washed 4 times with 0.1 M phosphate buffer (pH = 7.4), and postfixed with osmium tetroxide (Sigma-Aldrich, St. Louis, MO, USA). They were then dehydrated in a series of acetone solutions of increasing concentrations (30–100%) and infiltrated with EMbed 812 (Electron Microscopy Sciences, Hatfield, PA, USA). From the blocks obtained, 70 nm thick sections were cut on an LKB Ultrotome III Bromma 8800 ultramicrotome (LKB Produckter AB, Stockholm, Sweden) using a DiATOME diamond knife (DiATOME, Hatfield, PA, USA). Following their collection on 300 mesh copper grids (Agar Scientific Ltd., Stansted, UK), the sections were double contrasted with uranyl acetate and lead citrate. The samples were examined on a JEOL JEM 100CX II microscope (Jeol Ltd., Tokyo, Japan), at 80 kV, equipped with a MegaView G3 camera, operating with the Radius 2.1 software (both from Emsis, Münster, Germany).

#### 2.2.4. Tissue Processing for Biochemical Analyses

Liver tissue samples were homogenized using a POLYTRON PT 1200 E (Kinematica AG Luzemerstrasse 147a CH-6014, Lithau-Lucerne, Switzerland) homogenizer in TRIS buffer (50 mM) containing EDTA (10 mM), pH 7.5. Homogenates were centrifuged for 30 min at 10,000 rpm. Supernatant was collected and used for analyses. Protein concentration was determined by the Bradford method [25].

#### 2.2.5. Liver Function, Inflammatory and Fibrosis Markers

Aspartate aminotransferase (AST), alanine aminotransferase (ALT), cholesterol, glucose, and triglycerides, were determined from serum by using spectrophotometric methods with commercially available diagnostic kits (Hospitex Diagnostic, Sesto F.no-Firenze, Italy), following the manufacturer’s instructions.

The levels of pro-inflammatory cytokines interleukin 6 (IL-6), interleukin 1β (IL-1β), tumor necrosis factor α (TNF-α), and the fibrosis marker transforming growth factor beta (TGF-β) were measured from the liver lysates by using enzyme-linked immunosorbent assay (ELISA) kits, according to the manufacturers’ protocols (Elabscience, Wuhan, China). Results were expressed as pg/mg protein.

#### 2.2.6. Oxidative Stress Markers

Lipid peroxidation was evaluated by measuring malondialdehyde (MDA) levels using a thiobarbituric acid–based fluorimetric assay. Plasma samples and tissue homogenates were incubated with 2-thiobarbituric acid (10 mM) in K_2_HPO_4_ buffer (75 mM, pH 3), followed by extraction in n-butanol. Fluorescence was measured by synchronous spectrofluorimetry (Δλ = 14 nm) at 534 nm using Perkin Elmer LS 45 Fluorescence Spectrometer U.S (Waltham, Massachusets, USA), and MDA concentrations were expressed as nM/mg protein [26].

Reduced and oxidized glutathione (GSH and GSSG) levels were determined using a fluorimetric method based on o-phthalaldehyde derivatization. Fluorescence was recorded at an excitation wavelength of 350 nm and an emission wavelength of 420 nm, using Perkin Elmer LS 45 Fluorescence Spectrometer U.S [27].

Glutathione peroxidase (GPx) activity was spectrophotometrically assessed by monitoring NADPH consumption at 340 nm according to the method of Flohé et al. [28]. Superoxide dismutase (SOD) activity was measured in liver lysates using a commercial assay kit (Sigma-Aldrich), based on the inhibition of nitro blue tetrazolium reduction. Enzyme activities were expressed as enzymatic units/gram protein [29].

#### 2.2.7. Statistical Analysis

Data were analyzed using GraphPad Prism (v8.2; GraphPad Software, San Diego, CA, USA; downloaded on 15 March 2019) Normality was evaluated using the Shapiro–Wilk test. For normally distributed data, group differences were assessed by one-way ANOVA followed by Fisher’s LSD multiple-comparisons test. For non-normally distributed data, the Kruskal–Wallis test was applied, followed by Dunn’s multiple-comparisons test. Results are reported as mean ± SD. A two-sided *p*-value < 0.05 was considered statistically significant. Graphs were generated in GraphPad Prism (v8.2).

## 3. Results

### 3.1. Characterization of Rutin-Phytoreduced Gold Nanoparticles

Gold nanoparticles were synthesized via a green and facile reduction method using rutin as a natural reducing and capping agent. Upon mixing the rutin basic solution with the Au^3+^ solution, a rapid color change from pale yellow to intense purple red was observed, indicating that the reduction process had occurred. In addition to reducing Au^3+^ ions, rutin also acted as a stabilizing agent by binding to the nanoparticle surface through its hydroxyl functional groups, thereby preventing aggregation and yielding stable, well-dispersed gold nanoparticles.

The size and shape of gold nanoparticles (AuNPs) play a critical role in determining their biological activity, including cellular uptake, biodistribution, toxicity and therapeutic efficacy [30].

**Figure 2:**
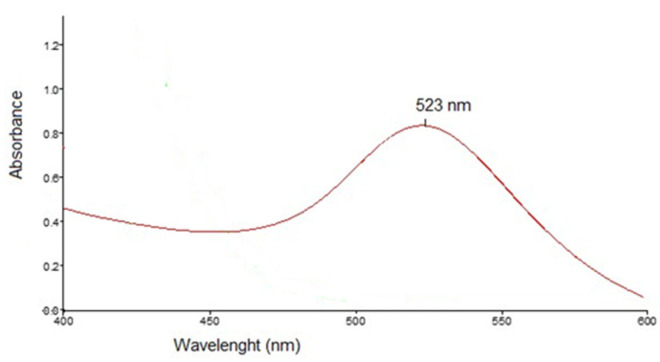
UV–Vis spectrum of the rutin-phytoreduced nanoparticles.

Characterization of the nanoparticles was further done by UV–Vis spectroscopy and transmission electron microscopy (TEM). The UV–Vis spectrum of rutin-reduced gold nanoparticles exhibited a characteristic absorption maximum at 523 nm, confirming the formation of gold nanoparticles (Figure 2).

**Figure 3:**
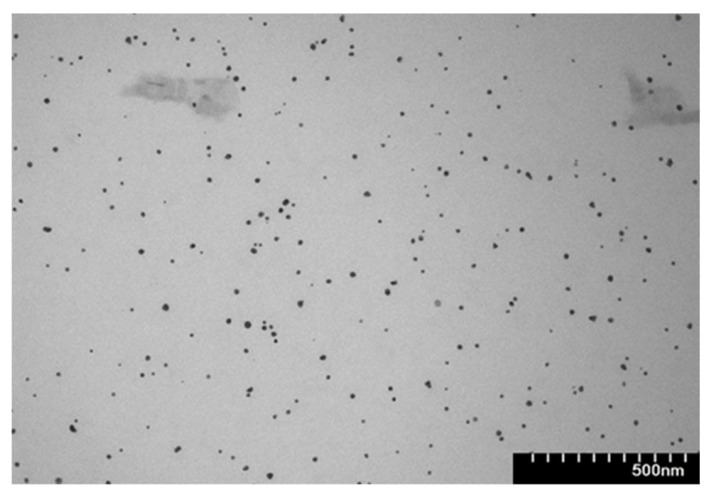
Transmission electron microscopy of the rutin-reduced gold nanoparticles.

The size, shape, and morphology of the synthesized gold nanoparticles were examined using transmission electron microscopy. The TEM image confirmed the formation of nearly spherical gold nanoparticles, with a narrow distribution and an average diameter of 18 nm (Figure 3).

**Figure 4:**
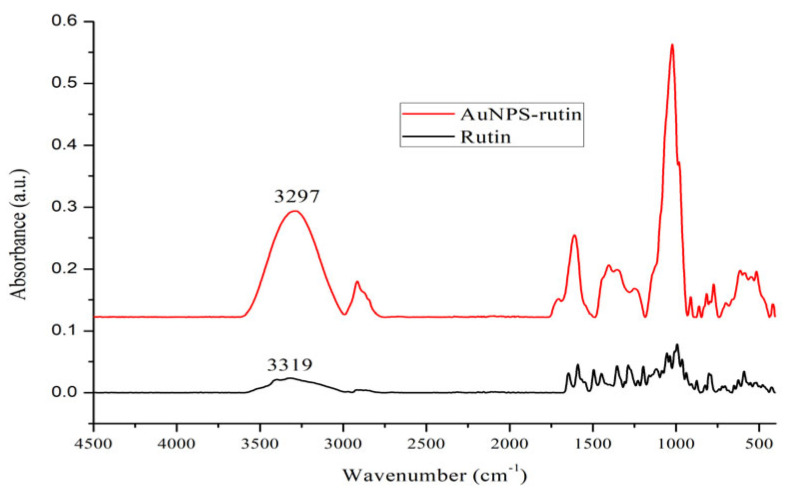
FTIR spectra of rutin and rutin-reduced gold nanoparticles.

The presence of the rutin at the surface of gold nanoparticles, acting as both capping and stabilizing agent, was confirmed by FTIR spectroscopy. The FTIR spectra of rutin and rutin-capped gold nanoparticles are presented in Figure 4. The spectrum of rutin shows characteristic absorption bands to OH stretching vibration around 3300–3400 cm^−1^, C=O stretching of the flavonoid ketone at 1647 cm^−1^. In the rutin-capped AuNPs spectrum, the OH and C=O stretching bands exhibit a slide shift to lower wavenumbers, indicating that these functional groups are involved in the stabilization of AuNPs, being capped at their surface.

**Figure 5:**
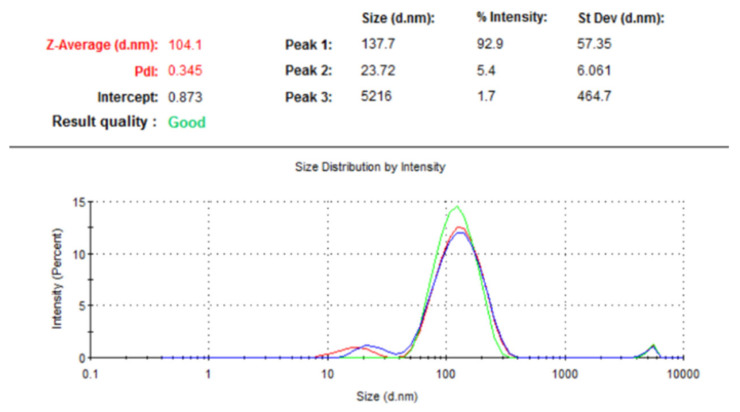
DLS analysis of rutin-capped gold nanoparticles.

Dynamic light scattering (DLS) measurements were performed to evaluate the hydrodynamic size and size distribution of the synthesized gold nanoparticles. These exhibited an average hydrodynamic diameter of 104.1 nm with a polidispersity index (PDI) of 0.345 (Figure 5). As expected, the hydrodynamic size is larger than the particle size observed in the TEM images, indicating the effective diameter of the particles, including the solved layer and the adsorbed rutin molecules. The PDI value indicates a moderately broad size distribution, suggesting some variation in particles’ size. Overall, the DLS results confirm that the synthesized gold nanoparticles are well-dispersed and the capping layer of rutin molecules contributes to their colloidal stability.

### 3.2. Morphological Liver Alterations

**Figure 6:**
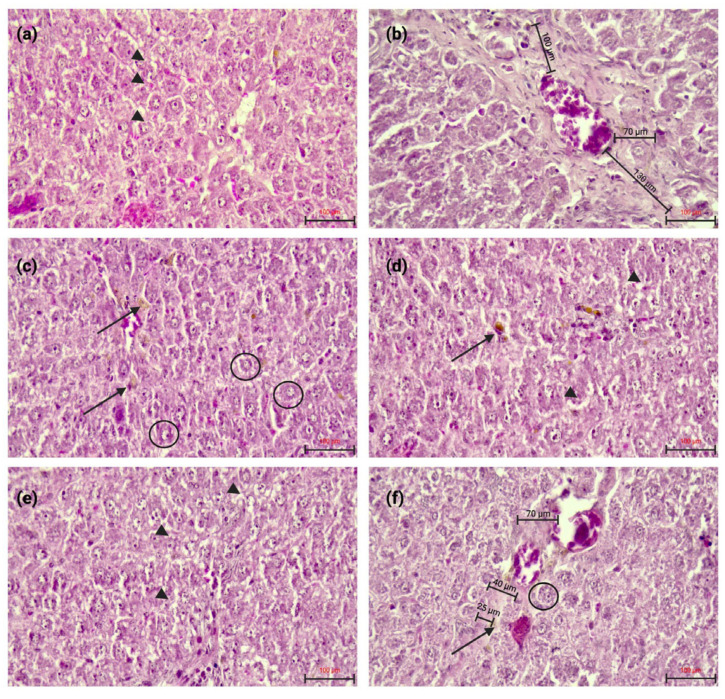
Representative H&E-stained liver sections from the experimental groups. (**a**) Control: preserved hepatic architecture. (**b**) TAA: increased vascular wall thickness; the measured segment is indicated by black line. (**c**) Ru: vacuolar degeneration (arrowheads) and hepatocellular pigment accumulation (black arrow). (**d**) TAARu: persistence of vacuolar degeneration and pigment deposits after TAA exposure. (**e**) RuAuNP: dystrophic changes with regenerative features (arrowheads). (**f**) TAARuAuNP: similar dystrophic/regenerative changes (arrow), with additional areas of interest highlighted (circles). Images are representative of each group. Scale bar: 100 μm.

Histological examination using hematoxylin–eosin staining (Figure 6) showed that, compared with the Control group, TAA increased the vascular wall thickness, a marker of the fibrogenic hepatic response. This increase was reflected by a higher mean vascular thickness (VT: 100 ± 24.5 µm), whereas VT was lower in the TAA + RuAuNP group (45 ± 18.7 µm) (Table 1). Evaluation using the Knodell histological activity index showed varying degrees of liver injury among groups. As a composite index, the Knodell score may not fully capture differences in lesion pattern (dystrophic injury versus fibrotic remodeling); therefore, we also report VT measurements and lesion/area estimates (Table 1). Rutin alone caused mild histological changes, mainly characterized by mixed dystrophy, predominantly vacuolar and pigment accumulation in hepatocytes. Combined treatment of TAA and rutin did not improve the TAA and rutin-induced alterations. In contrast, rutin-phytoreduced AuNPs (RuAuNP) induced dystrophic yet regenerative changes. These lesions are suggestive of osmotic-type hepatic alterations induced by the nanoparticles at the administered dose. Similar features were observed in the TAA + rutin-phytoreduced AuNP group. Importantly, the lesion pattern differed from TAA, with milder fibrosis in TAARuAuNP (~30% area) compared with TAA (~50% area), together with reduced vascular wall thickening (Table 1), supporting a beneficial effect of the nanoformulation on fibrotic progression.

**Figure 7:**
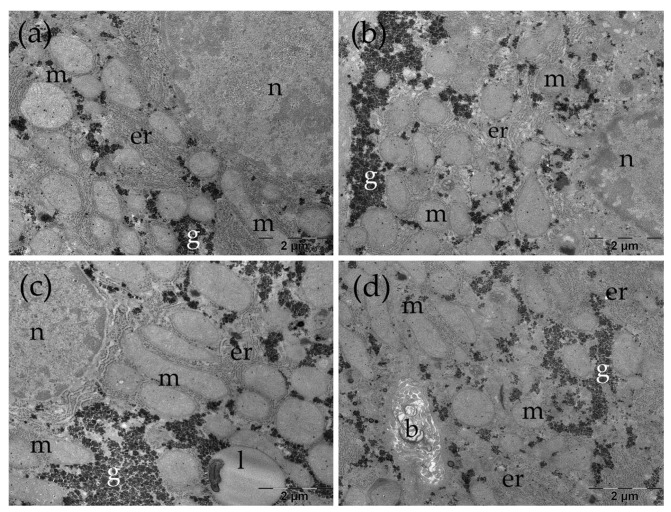
Ultrastructural several aspects of the liver in the Control group (**a**–**d**). b: bile canaliculus; er: endoplasmic reticulum; g: glycogen; l: lipid droplet; m: mitochondria; n: nucleus.

**Figure 8:**
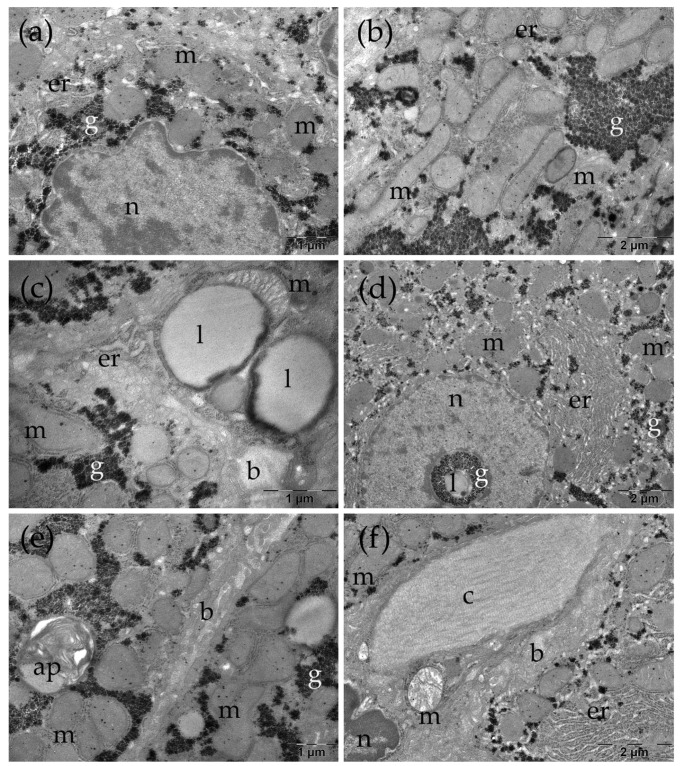
Ultrastructural several aspects of the liver in the TAA group (**a**–**f**). ap: autophagosome; b: bile canaliculus; c: collagen; er: endoplasmic reticulum; g: glycogen; l: lipid droplet; m: mitochondria; n: nucleus.

**Figure 9:**
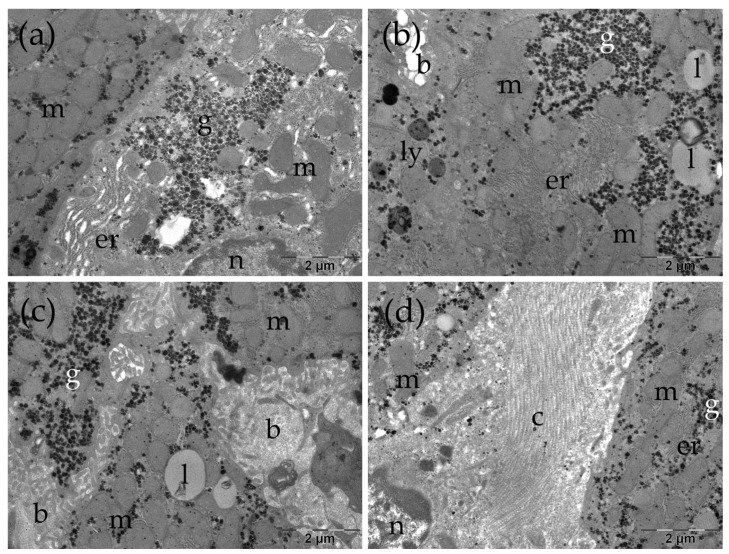
Ultrastructural several aspects of the liver in the TAA+rutin group (**a**–**d**). b: bile canaliculus; c: collagen; er: endoplasmic reticulum; g: glycogen; l: lipid droplet; ly: lysosomes; m: mitochondria; n: nucleus.

**Figure 10:**
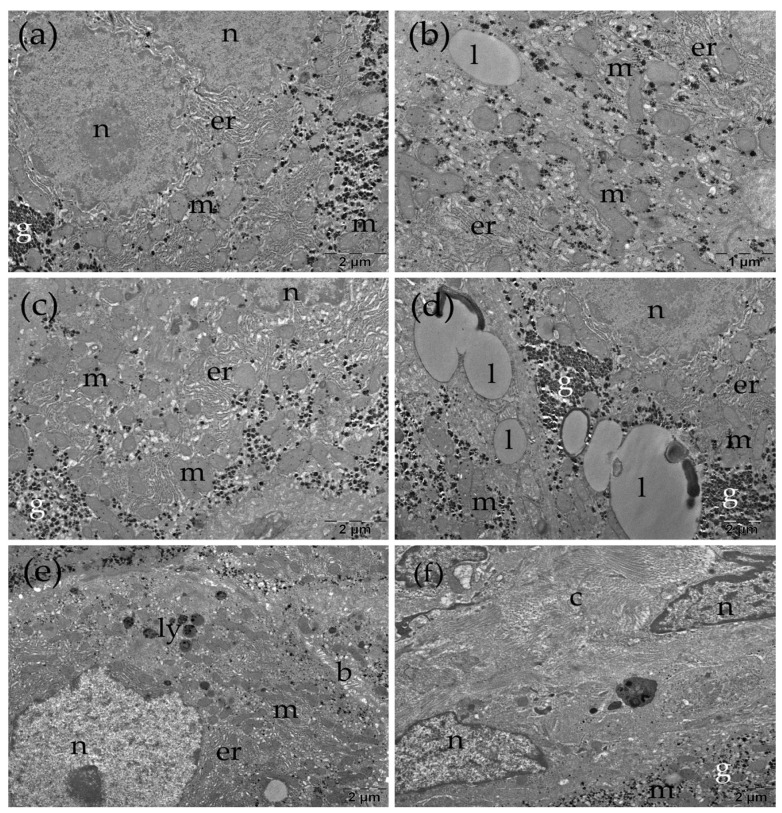
Ultrastructural several aspects of the liver in the TAA+NP group (**a**–**f**). b: bile canaliculus; c: collagen; er: endoplasmic reticulum; g: glycogen; l: lipid droplet; ly: lysosomes; m: mitochondria; n: nucleus.

Transmission electron microscopy (TEM) was employed as the method of choice for ultrastructural assessment, as it enables detailed visualization of intracellular organelles and subcellular architecture. Using TEM, we evaluated hepatocyte nuclear morphology, mitochondrial shape and organization, endoplasmic reticulum profiles, intracellular lipid and glycogen deposits, bile canaliculi integrity, and collagen-rich extracellular regions as ultrastructural correlates of injury and remodeling. Below, we describe representative ultrastructural findings in the Control, TAA, TAARu, and TAARuAuNP groups (Figure 7, Figure 8, Figure 9 and Figure 10).

In the Control group, the liver presented a normal ultrastructure. The hepatocytes had large, rounded nuclei and abundant cytoplasm (Figure 7a–c). The numerous mitochondria were round or oval, with small cristae (Figure 7a–d). Additionally, rough endoplasmic reticulum profiles and glycogen granules were observed (Figure 7a–d), alongside lysosomes or occasional small lipid droplets (Figure 7c), some with dense inclusions. Bile canaliculi with normal aspect were noted between adjacent cells (Figure 7d).

In the TAA group, many of the hepatocytes had irregular, indented nuclear contour (Figure 8a). Long mitochondria were observed in the cytoplasm (Figure 8b), as well as regions of expanded endoplasmic reticulum (Figure 8a,d) and occasional autophagosomes (Figure 8e). Large lipid droplets with an electron-dense outer rim were also noted (Figure 8c). Additionally, glycogen and lipid accumulations were present in some of the nuclei of the hepatocytes (Figure 8d). No changes were observed at the level of the bile canaliculi (Figure 8e).

In some areas, abundant collagen fibers were observed in the intercellular space (Figure 10f). The hepatocytes of the TAARu group had areas with expanded profiles of endoplasmic reticulum (Figure 9a). The lipid droplets were large and numerous, and some contained electron-dense inclusions (Figure 9b,c). No changes were observed at the level of the bile canaliculi (Figure 9c). Significant amounts of collagen fibers were also present between the cells in some regions (Figure 9d).

In the TAARuAuNP group, the hepatocytes had nuclei with slightly irregular contours (Figure 10a,c–e). Some of the mitochondria were long or polymorphic (Figure 10b,c), and expanded profiles of endoplasmic reticulum were observed (Figure 10a–e). Large lipid droplets were also noted, some with a heterogenous aspect (Figure 10c). No changes were observed at the level of the bile canaliculi (Figure 10e). Between the hepatocytes, there were collagen-abundant regions (Figure 10f).

To enable a consolidated comparison of ultrastructural changes, TEM findings were summarized using semiquantitative scoring across five domains (Table 2). Compared with Control, TAA showed lower scores for nuclear contour, ER profiles, lipid burden, and collagen. The TAARu group showed intermediate scores across most criteria. In contrast, the TAARuAuNP group exhibited the lowest scores in several domains, particularly for ER dilation and collagen/fibrosis, indicating more pronounced ultrastructural abnormalities in the evaluated TEM fields.

**Figure 11:**
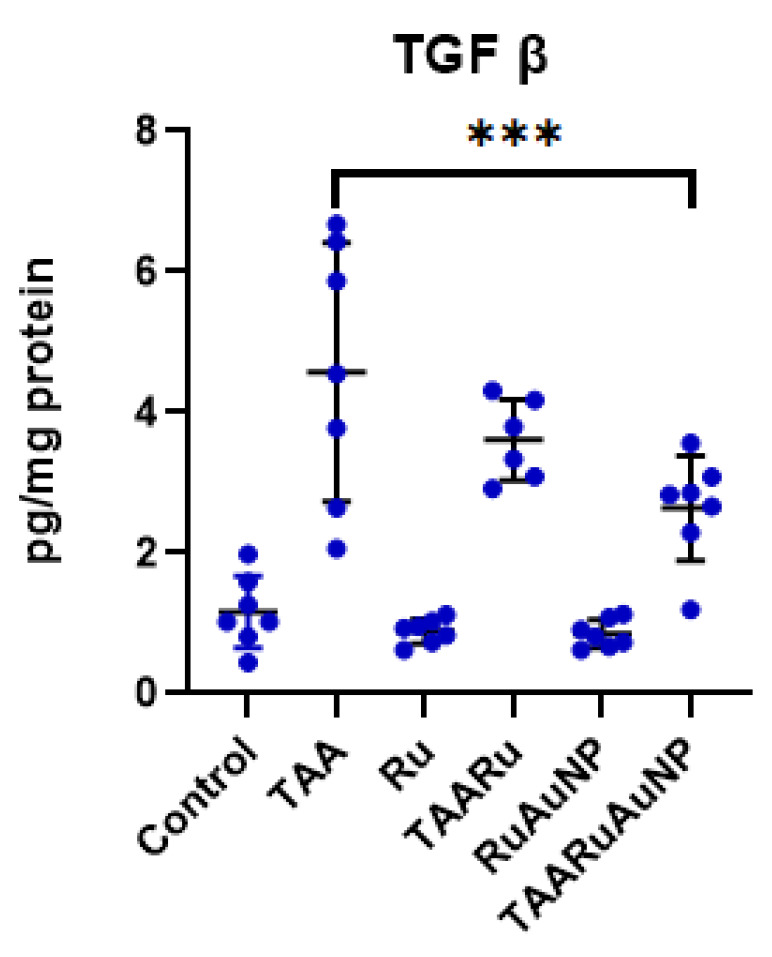
Hepatic TGF-β levels across experimental groups. Groups: Control, TAA, Ru, TAARu, RuAuNP, TAARuAuNP. Data are presented as mean ± SD (n = 7). *** *p* < 0.001.

Consistent with these findings, TGF-β levels (Figure 11) were strongly increased in the TAA group compared to Control (*p* < 0.0001), confirming activation of profibrotic pathways. TGF-β was also significantly higher than Control in both treated TAA groups (*p* ≤ 0.0034), indicating that fibrogenic signaling remained elevated despite treatment. TAARuAuNP significantly reduced TGF-β compared with the TAA fibrotic group (*p* = 0.0002), while TAARu exhibited no significance. Overall, AuNP-mediated delivery of rutin down-modulated TGF-β in the fibrotic setting, even though levels did not fully return to Control values within the experimental timeframe.

### 3.3. Liver Function and Metabolic Results

**Figure 12:**
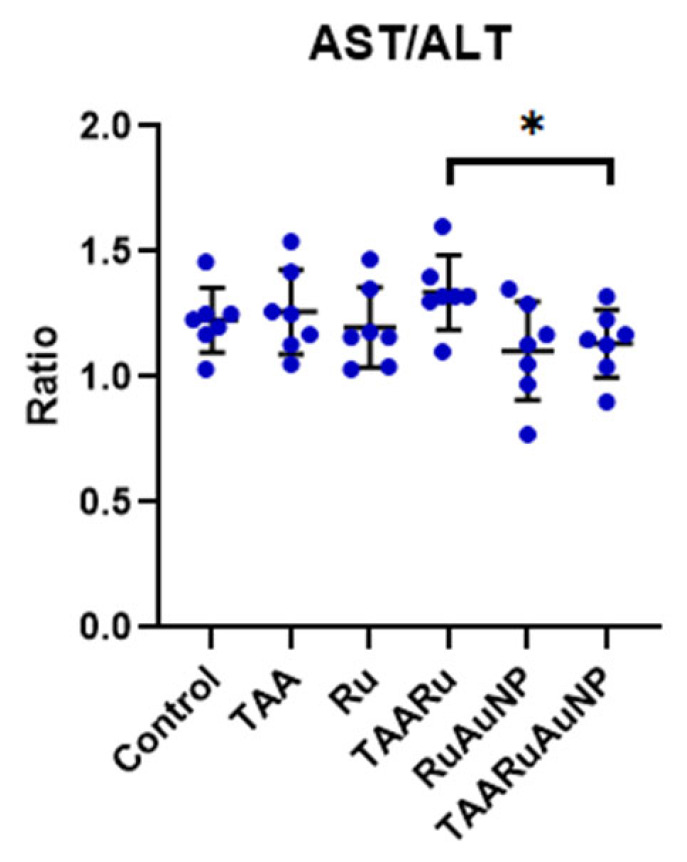
Liver transaminase (AST/ALT) ratios across experimental groups. Groups: Control, TAA, Ru, TAARu, RuAuNP, TAARuAuNP. Data are presented as mean ± SD (n = 7). Significance marks indicate comparisons versus the Control group * *p* < 0.05.

All animals completed the experimental protocol, and no mortality was recorded during TAA administration or the subsequent treatment period (0/42). The TAA group showed marked elevations of AST compared to Control (*p* < 0.0001), consistent with hepatocellular injury. AST levels remained similarly increased in the treated TAA groups, with no significant differences versus TAA (*p* ≤ 0.8129) (Table 3). In contrast, the AST/ALT ratio differed between treatments, being significantly lower in TAARuAuNP compared with TAARu (*p* = 0.0215), leading to partial normalization of the enzymatic profile with nanoparticle-mediated rutin delivery (Figure 12).

**Figure 13:**
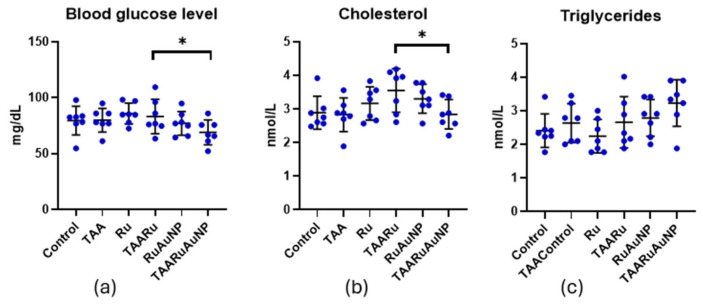
Metabolic parameters across experimental groups. (**a**) Blood glucose; (**b**) total cholesterol; (**c**) triglycerides. Groups: Control, TAA, Ru, TAARu, RuAuNP, TAARuAuNP. Data are presented as mean ± SD (n = 7). * *p* < 0.05.

Metabolic readouts showed limited changes attributable to TAA exposure (Figure 13). Blood glucose and cholesterol showed no significantly different levels between Control and TAA (*p* ≥ 0.7684). TAARuAuNP displayed significantly lower glycemia than TAARu (*p* = 0.0307), and Ru (*p* = 0.0118). For cholesterol, a significant difference was observed between TAARu and TAARuAuNP (*p* = 0.0268). Triglycerides were significantly lower in Control (*p* = 0.0155) and Ru (*p* = 0.0045) compared to TAARuAuNP.

### 3.4. Oxidative Stress

**Figure 14:**
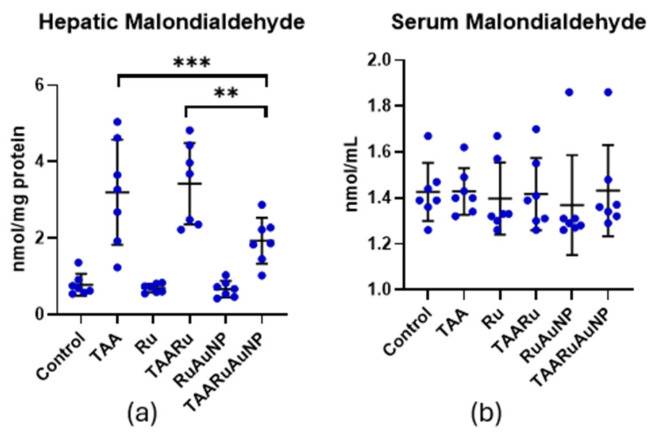
Hepatic and serum malondialdehyde (MDA) levels across experimental groups. (**a**) Hepatic MDA; (**b**) serum MDA. Groups: Control, TAA, Ru, TAARu, RuAuNP, TAARuAuNP. Data are presented as mean ± SD (n = 7). ** *p* < 0.01; *** *p* < 0.001.

Hepatic malondialdehyde (MDA), a marker of lipid peroxidation, was significantly increased after TAA exposure, compared to Control (*p* < 0.0001), confirming pronounced oxidative damage in liver tissue (Figure 14). In contrast, TAARuAuNP significantly lowered hepatic MDA compared with both the untreated fibrotic group and the free rutin group (*p* ≤ 0.0038), supporting a stronger antioxidant effect when rutin was delivered via AuNPs. Nevertheless, hepatic MDA in TAARuAuNP remained higher than Control (*p* = 0.0079), suggesting partial but incomplete recovery within the experimental timeframe.

Serum MDA did not show a consistent treatment- or TAA-related shift, with no significant differences between Control and any TAA group (*p* > 0.05), indicating that lipid peroxidation changes were primarily detected at the hepatic level in this model (Figure 14).

**Figure 15:**
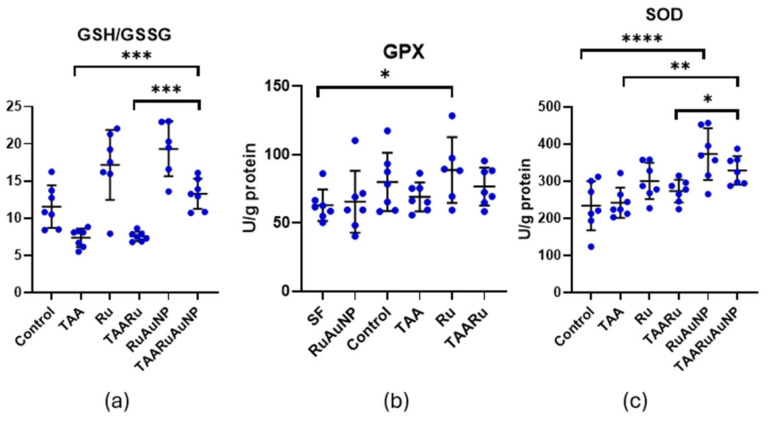
Hepatic antioxidant defense markers across experimental groups. (**a**) GSH/GSSG ratio; (**b**) glutathione peroxidase (GPx) activity; (**c**) superoxide dismutase (SOD) activity. Groups: Control, TAA, Ru, TAARu, RuAuNP, TAARuAuNP. Data are presented as mean ± SD (n = 7). * *p* < 0.05; ** *p* < 0.01; *** *p* < 0.001; **** *p* < 0.0001.

The GSH/GSSG ratio, a key indicator of glutathione-dependent redox balance, was significantly altered by TAA exposure versus Control (*p* = 0.0098), supporting the presence of oxidative stress in the fibrotic setting (Figure 15). Importantly, TAARuAuNP showed a significant improvement in this parameter compared with both the untreated fibrotic group and free rutin (*p* ≤ 0.0006), consistent with a stronger antioxidant effect when rutin was delivered via AuNPs. Moreover, the GSH/GSSG ratio in TAARuAuNP was not significantly different from Control (*p* = 0.2657), suggesting a shift towards redox normalization.

GPx activity showed limited modulation in this model (Figure 15). A significant difference was detected only under non-fibrotic conditions, Control versus Ru groups (*p* = 0.0144), while no significant differences were observed among TAA-exposed groups (*p* > 0.05). Overall, these results indicate that the nanoparticle induced antioxidant benefit was better captured by restoration of the glutathione redox system rather than by consistent changes in GPx activity.

Superoxide dismutase (SOD), a key enzymatic antioxidant involved in superoxide radical scavenging, showed a significant treatment effect in the fibrotic setting (Figure 15). TAARuAuNP exhibited higher SOD activity than the TAA untreated fibrotic group (*p* = 0.0029). TAARu exhibited no statistical significance as compared to TAA. Moreover, SOD activity was significantly higher in TAARuAuNP compared with TAARu (*p* = 0.0479), supporting an AuNP-dependent enhancement of enzymatic antioxidant defense.

Overall, these findings indicate that TAA-induced liver fibrosis was accompanied by oxidative stress and dysregulated antioxidant responses. Consistent with the MDA and glutathione data, rutin-phytoreduced AuNPs provided a more robust antioxidant signature than free rutin, including reduced lipid peroxidation, improved glutathione redox balance, and increased SOD activity, suggesting that nanoparticle-mediated delivery strengthens rutin’s cytoprotective effects on hepatic oxidative stress pathways.

### 3.5. Inflammatory Response

IL-1β and IL-6 are key pro-inflammatory cytokines. They are mainly produced by macrophages during oxidative stress exposure and hepatocellular injury. Therefore, their levels reflect the severity of inflammation in our model of the thioacetamide-induced liver fibrosis and the anti-inflammatory potential of treatments. TNF-α is another central mediator of hepatic inflammation, whose elevation indicates activation of inflammatory pathways in this model.

**Figure 16:**
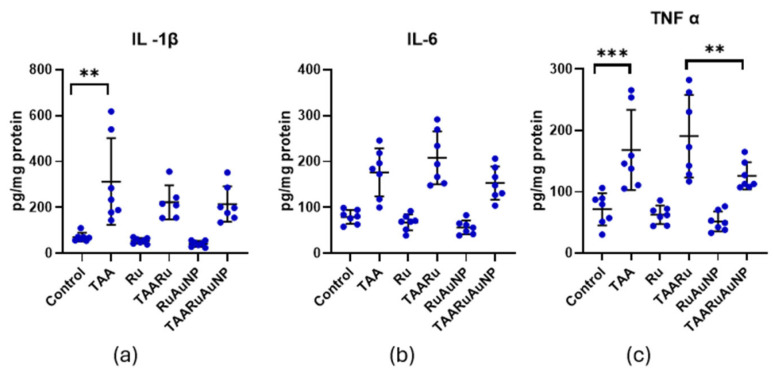
Hepatic pro-inflammatory cytokine levels across experimental groups. (**a**) IL-1β; (**b**) IL-6; (**c**) TNF-α. Groups: Control, TAA, Ru, TAARu, RuAuNP, TAARuAuNP. Data are presented as mean ± SD (n = 7). ** *p* < 0.01; *** *p* < 0.001.

TAA exposure induced a pronounced inflammatory response, as reflected by significantly higher IL-1β (*p* ≤ 0.0288), IL-6 (*p* ≤ 0.0006), and TNF-α (*p* ≤ 0.0201) levels in all TAA-treated groups compared with non-fibrotic controls (Figure 16). For IL-1β, no significant differences were observed among the TAA groups. Neither rutin nor rutin-phytoreduced AuNPs significantly modulated this cytokine relative to the untreated fibrotic group within the experimental timeframe. In contrast, both IL-6 and TNF-α showed significant differences between TAARu and TAARuNP (*p* ≤ 0.008). This suggests that rutin delivered via AuNPs induced a distinct improved inflammatory profile compared with free rutin, although neither treatment reached statistical significance versus TAA for these cytokines.

## 4. Discussion

This study evaluated whether delivering rutin via phytoreduced gold nanoparticles (RuAuNP) enhances the hepatic antifibrotic efficacy and biochemical profile in a thioacetamide-driven model of chronic liver injury.

Rutin was selected as treatment due to its well-described antioxidant and anti-inflammatory actions, which are directly relevant to mechanisms sustaining liver fibrogenesis. Its low oral bioavailability further justified evaluating whether nanoparticle-mediated delivery could enhance functional efficacy in vivo. Rutin’s mechanism of action is relevant to the antifibrotic profile observed and can be framed around two interconnected nodes: redox control and inflammatory signaling. In experimental liver injury, rutin has been reported to upregulate the Nrf2/HO-1 antioxidant program, strengthening endogenous defenses against ROS, while concurrently downregulating NF-κB–dependent inflammatory signaling and related mediators like TNF-α, COX-2, iNOS [31]. Because oxidative stress and NF-κB–driven inflammation promote hepatic stellate cell activation and extracellular matrix deposition, dampening these upstream drivers is mechanistically consistent with reduced profibrotic signaling and fibrogenic remodeling in chronic liver injury [32].

The TAA model is widely used because it generates sustained hepatocellular stress with intertwined oxidative and inflammatory responses that drive fibrogenic signaling [33,34]. In line with this framework, the TAA group showed clear histological injury and a marked increase of the hepatic TGF-β, supporting robust activation of profibrotic signaling. Importantly, TAARuAuNP significantly reduced TGF-β compared with TAA, whereas free rutin (TAARu) did not produce a comparable effect compared to the TAA group. This pattern is consistent with the central role of TGF-β in fibrogenesis and with prior TAA-induced fibrosis studies where attenuation of oxidative stress and inflammatory cascades converges on reduced TGF-β/Smad signaling and improved tissue architecture [35].

H&E sections showed prominent vascular and parenchymal alterations following TAA exposure, indicative of the induced liver damage. The nanoformulation yielded a qualitatively more favorable pattern than free rutin under TAA exposure. The presented data indicate that rutin, both in its free form and in its nanoformulated form, is bioactive, but not sufficiently to completely counteract the effects of TAA. However, rutin therapy appeared to halt the lesions at a predominantly dystrophic stage, without clear progression toward fibrotic processes. In addition, RuAuNP seemed more effective than free rutin, as reflected by the marked reduction in vascular wall thickening (##) compared with the TAA group. Taken together, these findings suggest that nanoformulated rutin represents a promising premise for further exploration in this field. The mild structural changes observed in RuAuNP alone can be interpreted in light of the known hepatic handling of AuNPs—preferential liver accumulation and predominant uptake by Kupffer cells—which may produce subtle exposure-related parenchymal alterations even in the absence of a co-administered hepatotoxic [36,37]. The presence of intranuclear glycogen, shown by TEM analysis has been correlated with numerous pathologies, such as diabetes, obesity, glycogen storage disorders or Wilson’s disease. The intranuclear lipids are also markers of hepatocyte damage [38].

The dystrophic pattern described as “osmotic-type” is consistent with vacuolar/hydropic change, a form of acute cell swelling that is widely regarded as a potentially reversible, sublethal response to cellular stress. [39]. This lesion aspect, named as “regenerative” reflects a pattern suggestive for adaptation/repair rather than progressive destruction. This is in line with prior AuNP liver reports, describing vacuolar-to-hydropic degeneration together with features such as binucleation, often discussed as a marker of hepatocyte turnover after injury [40].

In addition, the semiquantitative TEM summary (Table 2) indicated that TAARuAuNP displayed lower ultrastructural scores in several domains within the evaluated fields, particularly ER dilation and collagen-rich regions. This apparent mismatch with the more favorable tissue-level biochemical profile highlights an important limitation of TEM: it provides high-resolution information from selected micro-areas rather than whole-organ coverage [41]. Because liver injury in toxin models can be heterogeneous, sampled fragments may not fully represent the global organ status. Moreover, morphological alterations do not necessarily translate into proportional functional impairment, as hepatocellular metabolic and redox capacity can remain partially preserved despite subcellular remodeling. For this reason, we interpret ultrastructural findings in conjunction with integrative endpoints (histology, oxidative stress and inflammatory biomarkers, and profibrotic signaling), which better reflect the overall treatment effect at the organ level.

Although H&E does not directly quantify collagen deposition, the parallel reduction in TGF-β supports the interpretation that TAARuAuNP dampened profibrotic signaling and partially preserved liver structure. The combined evaluation of histology and TGF-β/Smad axis was previously used as supportive evidence of antifibrotic pathway modulation in TAA models. Dendropanax morbifera reduced TGF-β1/p-Smad2/3 alongside with improved histopathology and fibrosis-related readouts [35]. Dendropanoxide attenuated TAA-induced fibrosis with decreased TGF-β1/p-Smad2/3 and reduced collagen/α-SMA signals [42]. A comparable pattern was also reported for hesperidin in TAA-induced fibrosis, where antifibrotic effects were linked to modulation of TGF-β/α-SMA with supportive tissue-level improvement [15].

Although TAA-induced clear hepatocellular injury and profibrotic activation, its impact on systemic metabolic readouts was limited, as glucose and total cholesterol did not differ significantly between Control and TAA. In this context, the lower glycemia and cholesterol observed in TAARuAuNP compared with TAARu likely reflect a treatment-related modulation of metabolic homeostasis rather than a direct “reversal” of a pronounced TAA-driven dysmetabolic phenotype. This interpretation is consistent with the reported hypoglycemic and hypolipidemic properties of rutin [43], which have been linked to its antioxidant and anti-inflammatory actions and to improved metabolic regulation [17]. The nanoformulation likely enhanced the hepatic delivery and bioavailability of rutin, consistent with previously demonstrated nanomedicine strategies for liver targeting of rutin [44,45]. Triglycerides, however, displayed a variable pattern across TA groups. Since TAARuAuNP did not differ significantly from TAA or TAARu, we interpret these findings cautiously and consider triglycerides less sensitive to treatment effects in this protocol. Overall, these findings suggest that nanoparticle-mediated rutin delivery can beneficially modulate selected glycolipid readouts under hepatic injury conditions, with effects that vary across parameters, deserving further investigation.

Oxidative stress modulation was one of the clearest differentiators between formulations. Lipid peroxidation, reflected by hepatic MDA, is a well-established feature of TAA-driven liver injury and fibrosis, typically accompanied by depletion of antioxidant defenses such as GSH and reduced enzymatic activity (SOD) [42,46,47]. In our study, hepatic MDA was strongly increased after TAA and was significantly reduced by TAARuAuNP versus both TAA and TAARu. This indicates that nanoparticle-mediated delivery enhanced rutin’s antioxidant impact at tissue level. This direction is consistent with multiple TAA based fibrosis studies in which improved outcomes are closely linked to restoration of redox homeostasis, [42,46] proven by the reduced lipid peroxidation (lower MDA) together with recovery of glutathione-dependent and enzymatic antioxidant systems. Serum MDA did not show significant group differences in our experimental setting. This reflects localized intrahepatic oxidative damage and/or lower sensitivity of systemic MDA to the hepatic alterations.

The glutathione system provided a complementary readout of intracellular redox state. The GSH/GSSG ratio is widely used as an integrated indicator of glutathione-dependent antioxidant capacity; a lower ratio reflects a shift toward a more oxidized intracellular environment and reduced ability to buffer ROS [48,49]. In our data, TAARuAuNP differed significantly from TAA and TAARu, and it was not statistically different from Control, supporting a “toward-normalization” shift in glutathione redox balance with the nanoparticles. This aligns with TAA-induced fibrosis studies showing that effective interventions often improve oxidative injury by restoring glutathione-related defenses and antioxidant enzyme activity (e.g., GSH and SOD recovery with reduced lipid peroxidation) [42,50]. Consistent with this, SOD activity was higher in TAARuAuNP than in TAA, and respectively TAARu. This indicated improved capacity to detoxify superoxide radicals. Finally, reviews on liver fibrosis nanomedicine and flavonoid-based nanoformulations emphasize that successful systems frequently lead to improved antioxidant status (lower lipid peroxidation and improved glutathione/antioxidant metrics) as part of the antifibrotic efficacy [14,51].

Beyond rutin, several plant-derived treatments in TAA models report a similar antifibrotic pattern of histological improvement together with reduced profibrotic/oxidative signaling. Silymarin attenuated chronic TAA injury in mice and downregulated key fibrosis markers, including TGF-β1 and α-SMA, alongside with tissue-level improvement [52]. Dihydromyricetin administered in TAA-induced liver fibrosis in mice improved the histopathology aspect, and reversed oxidative damage leading to decreased hepatic MDA and increased antioxidant defenses such as SOD and GSH, together with dampening of inflammatory signaling [53]. A similar pattern co-modulation was reported for Gardeniae Fructus, which attenuated hepatic oxidation and inflammation in TAA-injected mice and was mechanistically linked to pathways including AMPK/SIRT1/NF-κB and Nrf2 signaling [54]. Comparable results have also been described for other dietary phytochemicals with antifibrotic profiles in TAA injury. In a rat TAA fibrosis setting, the flavone apigenin exhibited antifibrotic effects correlated with downregulation of TGF-β1 (along with other profibrotic signaling nodes), and simultaneous effects on oxidative stress and inflammation [55]. In addition, combining natural antioxidants has shown pathway-level convergence: pomegranate extract + curcumin in TAA-induced fibrosis in rats was reported to impact TGF-β/Smad3 and NF-κB signaling.

This reinforces the broader theme that plant-derived antioxidants often translate benefit through coordinated suppression of the inflammatory–oxidative loops that feed profibrotic signaling [56].

The effects induced by the RuAuNP are consistent with earlier AuNP-based strategies in liver injury. For example, silymarin-coated gold nanoparticles improved CCl_4_-induced hepatic injury in Wistar rats, with reductions in liver serum enzymes and fibrosis-related readouts, as indicated by histopathology and immunohistochemistry [57]. This type of AuNP–polyphenol combination is also discussed in liver-fibrosis nanotherapy reviews as a way to influence key fibrogenic compartments, including hepatic stellate cells and Kupffer cells [3]. As such we used a novel formulation of an AuNP platform delivery for an antioxidant—rutin with difficult oral absorption and liver accumulation to strengthen the tissue-level benefit compared with the free rutin alone in a TAA live fibrosis model. In a complementary toxicity-oriented setting, quercetin and arginine mitigated AuNP-induced hepatotoxicity in rats, improving hepatic oxidative injury, supporting the broader idea that flavonoids can counter oxidative damage in AuNP contexts [58]. Finally, recent reviews on liver fibrosis nanomedicine and flavonoid-based nanomedicines highlight that nanocarriers can enhance local exposure and cellular uptake, often translating into stronger antioxidant/anti-inflammatory effects with antifibrotic relevance [14,59,60].

Persistent AST/ALT elevation despite improvements in histology and mechanistic markers can be interpreted as an endpoint–timing mismatch rather than a contradiction. ALT and AST primarily reflect ongoing hepatocyte injury/necrosis and inflammation, not fibrosis burden per se. Therefore, they may remain elevated even when profibrotic signaling (e.g., TGF-β) and redox status begin to improve. This is particularly plausible in chronic toxin models such as TAA, which produce a predictable, sustained injury pattern with concurrent fibrosis development [61].

In our study, AST and ALT remained elevated at the end of the post-induction treatment phase despite clearer improvements in tissue-level readouts (histological directionality, reduced TGF-β, and improved redox markers). This pattern is plausible in the TAA model because aminotransferases primarily reflect ongoing hepatocyte membrane leakage/necrosis, while fibrosis regression and remodeling can persist for weeks after stopping the toxin and may not translate immediately into “normalized” enzymes. In support of this, TAA/TA (thioacetamide/thioacetamide analog) models explicitly describe that biochemical peaks can improve after drug withdrawal, while some liver enzymes can remain elevated, and that fibrotic injury is maintained for several weeks after discontinuation [62]. Therefore, in our dataset, the most conservative interpretation is that the RuAuNP produced a more robust shift in profibrotic/oxidative pathways, but the four-week treatment window after a six-week TAA induction likely captured a phase in which hepatocellular injury markers can lag behind pathway and structural improvements.

Liver targeting was inferred from the oral route and the known property of AuNPs to accumulate in the liver via RES/Kupffer cell uptake. However, dedicated biodistribution measurements are needed for direct confirmation [63].

To further strengthen these findings, future work could complement the current H&E-based assessment and mechanistic readouts with quantitative collagen-specific endpoints (e.g., Sirius Red morphometry or hydroxyproline content). Although the liver represents a preferential organ for gold nanoparticle accumulation, as shown by the hepatic induced effects, in the present study there were no pharmacokinetic or biodistribution experiments performed. Future studies are needed to quantify systemic exposure and organ targeting like ICP-MS-based Au measurements and cellular localization. Extending the follow-up period would also help clarify the durability of the antifibrotic response and whether longer treatment windows translate into more complete biochemical recovery.

## 5. Conclusions

TAA-induced consistent experimental liver fibrosis, supported by the histological injury, TEM morphology, increased hepatic TGF-β and oxidative and inflammatory changes. Compared to free rutin, the rutin-phytoreduced gold nanoparticles exhibited a significantly better antifibrotic profile, by decreasing TGF-β and the inflammatory changes and led to a more favorable tissue appearance on H&E and TEM sections. It also provided stronger antioxidant protection, with reduced hepatic oxidative damage and improved both antioxidant balance and antioxidant defense. Although AST and ALT remained elevated, the combined tissue and mechanistic data suggest that pathway and redox improvements may come before full biochemical recovery.

Overall, nanoparticle-mediated hepatic delivery of rutin significantly potentiated its therapeutic efficacy in the TAA-induced fibrosis model, supporting its potential use as an adjuvant strategy in the management of liver fibrotic diseases.

## Figures and Tables

**Table 1:** Summary of liver histopathology across experimental groups, including the Knodell score (mean ± SD), vascular wall thickness (VT, µm), and the predominant lesion type with the estimated affected area (%). VT = vascular thickness (µm); n = number of evaluated replicates.

Experimental Group	VT μm	Lesion/Area %	n	Knodell Score	Mean ± SD	*p* Value
**Control (a)**	-	-/0	3	1	1 ± 0.010	-
**TAA (b)**	100 ± 24.5	fibrosis/50	3	6	6 ± 0.050	***
**Ru (c)**	-	dystrophy/20	3	3	3 ± 0.018	**
**TAARu (d)**	-	dystrophy/30	3	10	10 ± 0.093	***/#
**RuAuNP (e)**	-	dystrophy/30	3	8	8 ± 0.085	**
**TAARuAuNP (f)**	45 ± 18.7	mild fibrosis/30	3	8	8 ± 0.085	***/##

Statistically significant differences between the groups (Knodell score) were calculated by using a Student’s *t*-test. Experimental groups were compared with the Control (vehicle) group; (significance is indicated as *) and respectively versus the TAA group, (significance is marked with #). *p* < 0.05 (/#), *p* < 0.01 (**/##), and *p* < 0.001 (***).

**Table 2:** Semi-quantitative TEM scoring of liver ultrastructure (10 = normal; 1 = abnormal) across experimental groups.

Group	Nuclear Contour	Polymorphic Mitochondria	Expanded ER	Lipids (Number/Size)	Fibrosis
Control	7.67 ± 0.58	8.33 ± 0.58	9.33 ± 0.58	9.00 ± 0.00	10.00 ± 0.00
TAA	5.00 ± 0.00 *	8.00 ± 1.00	6.33 ± 1.16	6.33 ± 0.58	7.00 ± 1.00
TAARu	6.33 ± 0.58	7.33 ± 0.58	8.33 ± 0.58	5.00 ± 1.00 *	6.67 ± 0.58
TAARuAuNP	4.33 ± 1.53 *	5.33 ± 0.58 *#	3.33 ± 0.58 **†	5.67 ± 1.16	3.67 ± 0.58 **

Data are presented as mean ± SD of three independent observers (n = 3). Group differences were analyzed using the Friedman test, matched by observer, followed by uncorrected Dunn’s post hoc test. * *p* < 0.05 and ** *p* < 0.01 vs. Control; # *p* < 0.05 vs. TAA; † *p* < 0.05 vs. TAARu.

**Table 3:** Levels of liver transaminases across the experimental groups.

Group	AST (U/I)	ALT (U/I)
Control	76.57 ± 5.966	62.70 ± 4.502
TAA	109.1 ± 8.977 ****	87.78 ± 11.69 ****
Ru	80.89 ± 9.961	67.54 ± 3.228
TAARu	107.8 ± 14.41	81.01 ± 10.23
RuAuNP	73.50 ± 7.652	67.69 ± 8.469
TAARuAuNP	102.7 ± 12.85	90.56 ± 4.738

Significance marks indicate comparisons versus the Control group **** *p* < 0.0001.

## Data Availability

All experimental data supporting the results is presented in the current article.

## Abbreviations

The following abbreviations are used in this manuscript:

α-SMA	Alpha-smooth muscle actin
Akt	Protein kinase B
ALT	Alanine aminotransferase
AMPK	AMP-activated protein kinase
ANOVA	Analysis of variance
ARRIVE	Animal Research: Reporting of In Vivo Experiments
AST	Aspartate aminotransferase
ATR	Attenuated total reflectance
AuNPs	Gold nanoparticles
b.w.	Body weight
COX-2	Cyclooxygenase-2
DLS	Dynamic light scattering
EDTA	Ethylenediaminetetraacetic acid
ELISA	Enzyme-linked immunosorbent assay
ER	Endoplasmic reticulum
FTIR	Fourier-transform infrared spectroscopy
GPx	Glutathione peroxidase
GSH	Reduced glutathione
GSSG	Oxidized glutathione
H&E	Hematoxylin and eosin
HAuCl_4_	Chloroauric acid
HO-1	Heme oxygenase-1
HSCs	Hepatic stellate cells
ICP-MS	Inductively coupled plasma mass spectrometry
IL-1β	Interleukin-1 beta
IL-6	Interleukin-6
iNOS	Inducible nitric oxide synthase
LPS	Lipopolysaccharide
LSD	Least significant difference
MAPK	Mitogen-activated protein kinase
MDA	Malondialdehyde
NADPH	Nicotinamide adenine dinucleotide phosphate (reduced form)
NF-κB	Nuclear factor kappa-B
Nrf2	Nuclear factor erythroid 2-related factor 2
PDI	Polydispersity index
PI3K	Phosphoinositide 3-kinase
RES	Reticuloendothelial system
ROS	Reactive oxygen species
Ru	Rutin
RuAuNP	Rutin-mediated gold nanoparticles
SD	Standard deviation
SIRT1	Sirtuin 1
Smad	Small mothers against decapentaplegic proteins
SOD	Superoxide dismutase
SPR	Surface plasmon resonance
TAA	Thioacetamide
TEM	Transmission electron microscopy
TGF-β	Transforming growth factor beta
TNF-α	Tumor necrosis factor alpha
TRIS	Tris(hydroxymethyl)aminomethane
UV–Vis	Ultraviolet–visible spectroscopy
VT	Vascular thickness
p-Smad2/3	Phosphorylated Smad2/3
